# Prefrontal Neuronal Excitability Maintains Cocaine-Associated Memory During Retrieval

**DOI:** 10.3389/fnbeh.2018.00119

**Published:** 2018-06-14

**Authors:** James M. Otis, Michael K. Fitzgerald, Hanna Yousuf, Jake L. Burkard, Matthew Drake, Devin Mueller

**Affiliations:** ^1^Department of Psychology, University of Wisconsin-Milwaukee, Milwaukee, WI, United States; ^2^Department of Psychiatry, University of North Carolina-Chapel Hill, Chapel Hill, NC, United States; ^3^Department of Basic Sciences, Ponce Health Sciences University-School of Medicine/Ponce Research Institute, Ponce, Puerto Rico

**Keywords:** intrinsic excitability, memory retrieval, cocaine abuse, PKA, norepinephrine, conditioned place preference (CPP), electrophysiology, medial prefrontal cortex (mPFC)

## Abstract

Presentation of drug-associated cues provokes craving and drug seeking, and elimination of these associative memories would facilitate recovery from addiction. Emotionally salient memories are maintained during retrieval, as particular pharmacologic or optogenetic perturbations of memory circuits during retrieval, but not after, can induce long-lasting memory impairments. For example, in rats, inhibition of noradrenergic beta-receptors, which control intrinsic neuronal excitability, in the prelimbic medial prefrontal cortex (PL-mPFC) can cause long-term memory impairments that prevent subsequent cocaine-induced reinstatement. The physiologic mechanisms that allow noradrenergic signaling to maintain drug-associated memories during retrieval, however, are unclear. Here we combine patch-clamp electrophysiology *ex vivo* and behavioral neuropharmacology *in vivo* to evaluate the mechanisms that maintain drug-associated memory during retrieval in rats. Consistent with previous studies, we find that cocaine experience increases the intrinsic excitability of pyramidal neurons in PL-mPFC. In addition, we now find that this intrinsic plasticity positively predicts the retrieval of a cocaine-induced conditioned place preference (CPP) memory, suggesting that such plasticity may contribute to drug-associated memory retrieval. In further support of this, we find that pharmacological blockade of a cAMP-dependent signaling cascade, which allows noradrenergic signaling to elevate neuronal excitability, is required for memory maintenance during retrieval. Thus, inhibition of PL-mPFC neuronal excitability during memory retrieval not only leads to long-term deficits in the memory, but this memory deficit provides protection against subsequent cocaine-induced reinstatement. These data reveal that PL-mPFC intrinsic neuronal excitability maintains a cocaine-associated memory during retrieval and suggest a unique mechanism whereby drug-associated memories could be targeted for elimination.

## Introduction

Presentation of environmental stimuli that predict drug availability can drive compulsive drug seeking and taking (Childress et al., 1986; Heather et al., 1991), making recovery from addiction difficult. Elimination of the memories that underlie stimulus-induced drug seeking would facilitate recovery from addiction, although the neurobiological mechanisms that maintain drug-associated memories are not clear. Past research reveals that the prefrontal cortex (PFC) engages drug seeking during the presentation of drug-associated cues. For example, in human addicts, presentation of drug-associated cues activates the PFC, and the level of PFC activation correlates with drug cravings (Grant et al., 1996; Kilts et al., 2001; Grüsser et al., 2004). In rodents, activity in prelimbic medial PFC (PL-mPFC) is important for cue-induced reinstatement of drug seeking, and particularly activation of PL-mPFC neurons that project to the nucleus accumbens (NAc; McFarland and Kalivas, 2001; McFarland et al., 2003; Stefanik et al., 2013; Ma et al., 2014; Pascoli et al., 2014). PL-mPFC cells that project to NAc are cue-responsive layer V pyramidal neurons (Otis et al., 2017), and these neurons undergo synaptic potentiation following drug experience (Robinson and Kolb, 1999, 2004; Robinson et al., 2001). Furthermore, this plasticity correlates with the acquisition of cocaine-associated memories *in vivo* (Muñoz-Cuevas et al., 2013). Recently, we found that drug-associated memory and synaptic plasticity in PL-mPFC pyramidal neurons are malleable, as inhibition of noradrenergic signaling during retrieval of a cocaine-associated memory not only leads to long lasting memory impairments (Otis and Mueller, 2011, 2017; Otis et al., 2013, 2014b; Fitzgerald et al., 2016), but also reverses cocaine-evoked synaptic plasticity in PL-mPFC pyramidal neurons (Otis and Mueller, 2017). Collectively, these data reveal that synaptic plasticity in layer V pyramidal neurons in PL-mPFC underlies drug-associated memories; however, how *intrinsic* plasticity might also contribute to drug-associated memories remains unclear.

In addition to synaptic plasticity, the activity of neural networks can be shaped through experience-dependent changes in intrinsic neuronal excitability (Woody and Black-Cleworth, 1973; Alkon, 1979; Disterhoft et al., 1986). Intrinsic plasticity can be altered long term, through experience-dependent modifications in ion-channel distribution and conductance, and short term, primarily through the actions of neuromodulators such as norepinephrine (NE) that rapidly influence ion channel conductance (Daoudal and Debanne, 2003; Nadim and Bucher, 2014; Otis et al., 2015). These long-term and short-term plasticity mechanisms are thought to underlie memory across multiple paradigms and within many brain regions (Zhang and Linden, 2003; Sehgal et al., 2013). Although not yet tested, this may be true for drug-associated memories as drug experience is known to cause long-term elevations in the intrinsic excitability of PL-mPFC pyramidal neurons (Dong et al., 2005; Nasif et al., 2005a,b, 2011; Hearing et al., 2013; Sepulveda-Orengo et al., 2018). In addition, NE, a neuromodulator that rapidly elevates PL-mPFC intrinsic neuronal excitability, acts in PL-mPFC to allow retrieval of cocaine-associated memories (Otis et al., 2013). However, how long-term and short-term intrinsic plasticity in PL-mPFC might contribute to the maintenance of drug-associated memories has not been determined.

Here, we evaluate how intrinsic plasticity in PL-mPFC pyramidal neurons maintains a drug-associated memory during retrieval. We first use patch-clamp electrophysiology to show that cocaine place conditioning, a model of drug-associated memory, leads to long-lasting elevations in the intrinsic excitability of PL-mPFC pyramidal neurons. Importantly, the long-term intrinsic plasticity correlates with conditioned place preference (CPP) memory retrieval, suggesting that it is a mechanism of retrieval. In addition to long-term intrinsic plasticity, we find that NE provokes rapid elevations in intrinsic excitability of PL-mPFC pyramidal neurons through cyclic AMP (cAMP)-dependent slow afterhyperpolarization (sAHP) inhibition. Furthermore, inhibition of this signaling cascade *in vivo* not only prevents memory retrieval during a single test, but also leads to long-term deficits in retrieval that prevent subsequent cocaine-induced reinstatement of the CPP. Overall, these data reveal that elevation of PL-mPFC intrinsic neuronal excitability at the time of retrieval is a likely mechanism that maintains cocaine-associated memories.

## Materials and Methods

### Subjects

Male Long-Evans rats (3–5 months, Harlan Laboratories, Indianapolis, IN, USA) were individually housed with unlimited access to water and rat chow. Rats were maintained on a 14-h light/10-h dark schedule (lights on at 7:00 am), and all behavioral protocols were completed during the light cycle. Surgeries were performed as previously described (Otis et al., 2013). Briefly, following anesthetization (90.5 mg/kg ketamine, 10.5 mg/kg xylazine, i.p.), a chronic indwelling double-barrel guide cannula (Plastics One) was implanted immediately dorsal to PL-mPFC (+2.8 mm anterior, ±0.6 mm lateral, −2.9 mm ventral relative to Bregma). Following surgery, rats were injected with an analgesic (carprofen, 5 mg in 0.1 mL, s.c.) and antibiotic (penicillin G, 75,000 units in 0.25 mL, s.c.). Stylets remained within cannula until microinfusions were performed to maintain patency. Rats were allowed to recover for a minimum of 7 days before all behavioral experiments. This study was carried out in accordance with the recommendations of the National Institutes of Health. The protocol was approved by the Institutional Animal Care and Use Committee at the University of Wisconsin-Milwaukee.

### Conditioning and Testing

Cocaine CPP experiments were performed as described previously (Otis and Mueller, 2011). Briefly, the apparatus contained three chambers wherein a small center chamber separated two distinguishable conditioning chambers. During CPP testing, rats had access to all three conditioning chambers for 15 min. We tested rats prior to conditioning and confirmed that no initial bias was present for either conditioning chamber across groups. Next, we used an unbiased procedure wherein rats were assigned to receive cocaine in one chamber, and saline in the other, in a pseudorandom and counterbalanced manner. Rats were conditioned during eight daily sessions, wherein cocaine or saline were administered in an alternating manner immediately before exposure to each conditioning chamber for 20-min sessions. Two days following the final conditioning trial, rats were given daily CPP tests, identical to tests prior to conditioning but with injections of saline 15 min before each test. For patch-clamp electrophysiology experiments, rats were sacrificed 1 h after the second CPP test. For microinfusion experiments, rats were given PL-mPFC microinfusions 30-min before the first CPP test, and no microinfusions during the second or third tests. Place conditioning data were analyzed by comparing time within each chamber across tests using three-way analysis of variance (ANOVA; all chambers, all groups, all tests). Following a significant main effect, two-way ANOVAs for each group were performed across tests (cocaine vs. saline chambers, all tests). Following a significant effect of chamber, Tukey’s *post hoc* tests were performed to compare time in the saline- vs. cocaine-paired chambers for each test.

To assess cocaine-induced reinstatement, following the initial CPP tests rats underwent further testing until extinction criterion was met (no preference for the cocaine vs. saline-paired chamber for each group for two consecutive tests). Rats then had a final extinction test followed by a cocaine-induced reinstatement test wherein a priming injection of cocaine (5 mg/kg, i.p.) was administered 15-min before testing. Reinstatement data were analyzed using a three-way ANOVA (all chambers, all groups, all tests), and because a change in behavior was expected between tests, following a significant main effect planned comparisons were performed to compare time in the saline- vs. cocaine-paired chambers during the reinstatement test for each group.

### Drugs and Microinfusions

Cocaine HCl was dissolved in sterile saline (0.9% NaCl) and was systemically administered at a dose of 10 mg/kg (i.p.) for behavioral conditioning and 5 mg/kg (i.p.) for reinstatement testing. The inhibitor of cAMP-dependent signaling Rp-2’-O-MB-cAMPs (BIOLOG), the sAHP inhibitor UCL-2077 (Tocris), and the MAPK antagonist U0126 (Tocris) were dissolved in equal parts sterile saline (0.9% NaCl) and dimethyl sulfoxide and were infused bilaterally at 3.33 μg/0.3 μl (Ouyang et al., 2008), 0.67 μg/0.3 μl (Zhang et al., 2013) and 1.33 μg/0.3 μl (Schafe et al., 2000), respectively. Following completion of microinfusion experiments, cannulae placements were histologically verified to be immediately dorsal to PL-mPFC (Supplementary Figure S1).

### Electrophysiology

Patch-clamp electrophysiological recordings were performed as described previously (Otis et al., 2013, 2014a). Briefly, rats were anesthetized with pentobarbital (50 mg/kg, i.p.) and brains were extracted and sectioned using a vibrating blade (Leica, VT1200) in ice-cold, oxygenated 95%O_2_:5%CO_2_) artificial cerebral spinal fluid (aCSF; in mM: 124 NaCl, 2.8 KCl, 1.25 NaH_2_PO_4_, 2 MgSO_4_, 2 CaCl_2_, 26 NaHCO_3_ and 20 glucose). Following sectioning, slices were incubated in warm aCSF (32°C) for a minimum of 1 h before electrophysiological experiments. During the experiments, brain sections were perfused constantly in the presence of picrotoxin (100 μM in aCSF, 32°C). Differential interference contrast microscopy (Nikon FN-1) was used to visualize layer V PL-mPFC pyramidal neurons, which were putatively identified based on shape and electrophysiological characteristics as previously described (Otis et al., 2013). Patch-clamp recordings were obtained using borosilicate glass pipettes (2–5 MΩ) containing an internal solution composed of the following (in mM: 110 K-gluconate, 20 KCl 10 HEPES, 2 MgCl_2_, 2 ATP, 0.3 GTP 10 phosphocreatine, 0.2% biocytin; pH 7.3 and mOsm 280). Data were collected using a signal amplifier (MultiClamp 700B, Molecular Devices) and digitizer (Digidata 1440A, Molecular Devices).

Current-clamp recordings were performed to quantify the intrinsic excitability of PL-mPFC pyramidal neurons in cocaine place conditioned rats. After recording the resting membrane potential of each neuron, slow polarizing current was applied to hold the neurons at −70 mV after subtraction of the liquid-liquid junction potential of 13 mV. Next, input resistance and rheobase were measured by applying 1-s steps until a single action potential was evoked (+10 pA steps, starting from −40 pA). Action potential frequency was evaluated through larger depolarizing steps (+50 pA steps, starting from 0 pA). Finally, we recorded action potential amplitude attenuation by adjusting the 1 s sweep amplitude until about 15 action potentials were evoked. To quantify action potential amplitude attenuation, we compared the amplitudes of the last vs. the first action potential.

Current-clamp recordings were also used to evaluate the mechanism whereby NE increases the intrinsic excitability of PL-mPFC pyramidal neurons. Specifically, while neurons were held at resting membrane potential, a 1-s current step was applied to evoke a baseline number of action potentials (generally 2–3 action potentials per sweep). After 2-min of stable recordings, NE (1 μM; Tocris) was bath applied within the aCSF for 2-min while recordings continued. For neurons in which cAMP-dependent processes were inhibited, Rp-2’-O-MB-cAMPs (500 μM; BIOLOG) was included within the internal solution of the patch pipette. Following collection, data was analyzed using Clampfit 10.3 (Molecular Devices), and ANOVA was used to compare data across groups. Following a significant main effect, each group of neurons was compared using planned comparisons.

## Results

### Intrinsic Excitability of PL-mPFC Pyramidal Neurons Is Elevated in Rats That Show Cocaine-Associated Memory Retrieval

**Figure 1:**
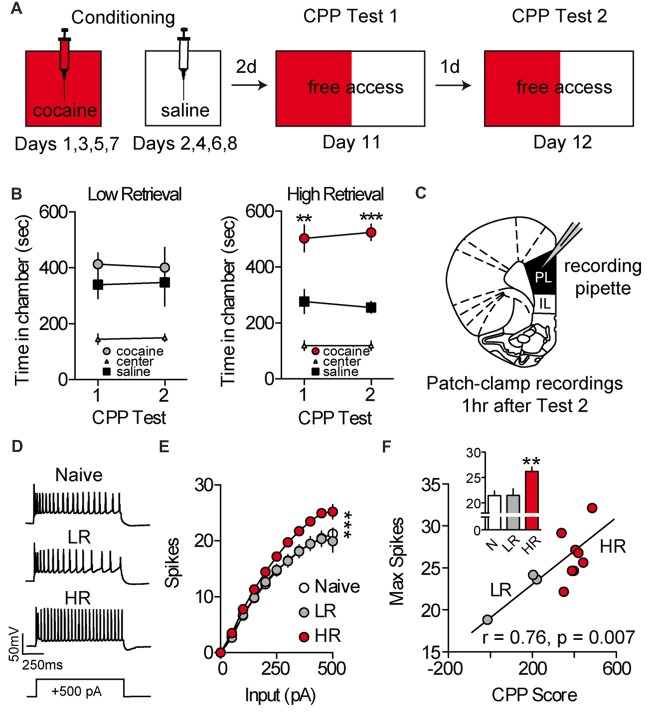
Intrinsic excitability of prelimbic medial prefrontal cortex (PL-mPFC) pyramidal neurons is increased in HR rats. **(A)** Schematic showing design of conditioned place preference (CPP) conditioning and testing. **(B)** Line graphs revealing that LR rats did not express a cocaine CPP during first or second tests, whereas HR rats did express a cocaine CPP during both tests. **(C)** Patch-clamp recordings of layer V pyramidal neurons in PL-mPFC were taken from rats sacrificed 1 h after the second CPP test. **(D)** Representative waveforms showing 500 pA depolarizing sweeps from layer V pyramidal neurons. **(E)** Neurons from HR rats displayed more spikes at high depolarizing steps as compared with naïve and LR rats. **(F)** The maximum number of spikes that were fired on average in PL-mPFC pyramidal neurons from each rat correlated with CPP scores. Inset shows that the maximum number of action potentials that neuron would fire at any depolarizing step was higher in HR rats vs. neurons from naïve and LR rats. Line and bar graphs represent the mean ± SEM. HR, high retrieval; LR, low retrieval; **p < 0.01 vs. control; ***p < 0.001 vs. control.

To evaluate the effects of cocaine place conditioning on intrinsic excitability of PL-mPFC pyramidal neurons, rats were given daily conditioning sessions wherein one chamber, but not another, was paired with cocaine (Figure 1A). Following conditioning, rats were given two daily CPP retrieval tests consisting of full access to all chambers. Overall, cocaine-treated rats (cocaine, *n* = 11; naïve, *n* = 5) had heterogeneous preferences for the previously cocaine-paired chamber vs. saline-paired chamber during these tests. Using a mean split, we categorized rats as having high retrieval (HR) scores (i.e., more time than average spent in the cocaine vs. saline-paired chamber; HR) or low retrieval (LR) scores (i.e., less time than average spent in the cocaine vs. saline-paired chamber; LR; Figure 1B). Next, naïve, LR and HR rats were sacrificed for patch-clamp electrophysiological recordings (Figure 1C). Recordings revealed that intrinsic excitability was increased in PL-mPFC pyramidal neurons from HR rats (*n* = 22 neurons), but not LR rats (*n* = 14 neurons), as compared with naïve rats (*n* = 14 neurons; Figures 1D,E). Two-way ANOVA revealed an input by group interaction (*F*_(20,470)_ = 3.535; *p* < 0.001), and planned comparisons revealed that significantly more spikes were evoked in neurons from HR rats as compared with neurons from naïve rats (sweeps between 0 pA and 150 pA, *p*s > 0.10; sweeps between 200 pA and 500 pA, *p*s < 0.05), whereas equivalent spikes were evoked in neurons from LR rats as compared with naïve rats (all sweeps, *p*s > 0.514). Consistent with this, the maximal firing frequency was greater in neurons from HR rats, but not LR rats, as compared with naïve (Figure 1F, inset), and this capacity for spiking correlated with cocaine CPP scores (Figure 1F; Pearson’s Correlation: *r* = 0.76, *p* = 0.007). One-way ANOVA revealed an effect of group for maximal firing frequency (*F*_(2,47)_ = 8.328, *p* < 0.001), and planned comparisons revealed that maximal firing frequency was higher in neurons from HR rats as compared with neurons from naïve rats (*p* < 0.001), whereas equivalent spikes were evoked in neurons from LR rats as compared with naïve rats (*p* = 0.964). Thus, cocaine conditioning increases the intrinsic excitability of PL-mPFC pyramidal neurons in HR rats, but not LR rats, although the mechanisms for these changes are unclear.

**Figure 2:**
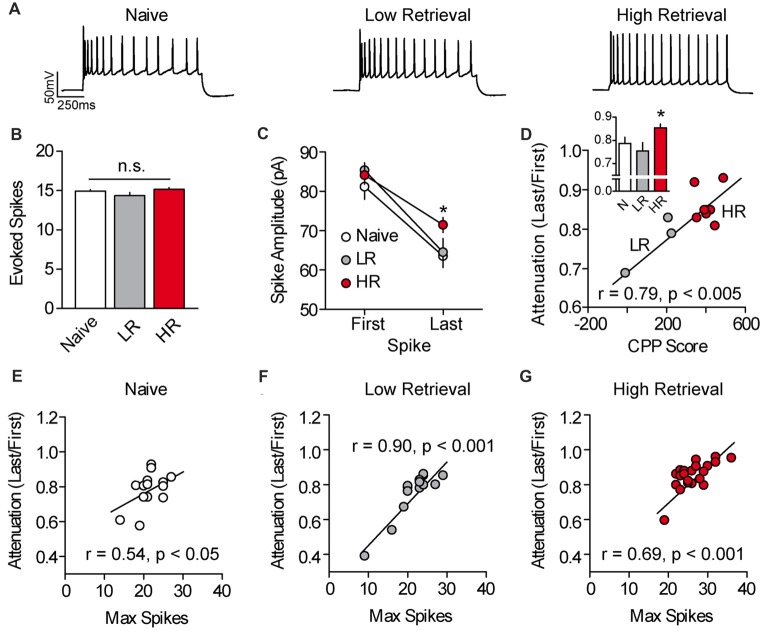
Spike amplitude attenuation of PL-mPFC pyramidal neurons is reduced in HR rats and correlates with intrinsic excitability. **(A)** Representative waveforms showing a current-evoked depolarizing step. **(B)** Current was adjusted so that roughly 15 action potentials fired per sweep. **(C)** The amplitude of the last, but not first, action potential had higher amplitude in neurons from HR rats vs. neurons from naïve and LR rats. **(D)** Spike amplitude attenuation in PL-mPFC pyramidal neurons from each rat correlated with CPP scores. Inset shows that spike amplitude attenuation was reduced in HR rats vs. neurons from naïve and LR rats. **(E–G)** Spike amplitude attenuation correlated with the maximum number of action potentials fired in each neuron for naïve rats **(E)**, LR rats **(F)** and HR rats **(G)**. Line and bar graphs represent the mean ± SEM. HR, high retrieval; LR, low retrieval; *p < 0.05 vs. control.

We next evaluated the mechanism of cocaine-associated intrinsic plasticity in PL-mPFC pyramidal neurons. We found that the intrinsic plasticity in HR rats could not be attributed to canonical adaptations in intrinsic excitability, as basic membrane properties and action potential properties were unchanged following conditioning (Supplementary Table S1). However, a previous study found that cocaine exposure can lead to an elevation of L-type calcium channel conductance in PL-mPFC pyramidal neurons (Nasif et al., 2005a), and such voltage-dependent calcium conductance has been shown to reduce spike amplitude attenuation and failure across spikes (Klyachko et al., 2001). Thus, we evaluated spike amplitude attenuation in PL-mPFC pyramidal neurons by evoking ~15 action potentials in each neuron using a current-adjusted depolarizing sweep (Figures 2A,B). Recordings revealed that the last spike, but not first spike, was of higher amplitude in PL-mPFC pyramidal neurons from HR rats (*n* = 22 neurons), but not LR rats (*n* = 14 neurons), as compared with naïve rats (*n* = 14 neurons; Figure 2C). ANOVA revealed a spike (two spikes, first and last) by group interaction (*F*_(2,47)_ = 3.884, *p* = 0.028), and planned comparisons revealed that the amplitude of the first action potential was not different between groups (*p*s > 0.273). In contrast, the amplitude of the last action potential was higher in neurons from HR rats as compared with neurons from naïve rats (*p* = 0.0251), whereas no significant difference was present in neurons from LR rats as compared with naïve rats (*p* = 0.0842). Thus, there was significantly reduced spike amplitude attenuation in neurons from HR rats as compared with naïve and LR rats (Figure 2D, inset), such that spike amplitude attenuation correlated with cocaine CPP scores (Figure 2D, Pearson’s Correlation: *r* = 0.785, *p* = 0.004). One-way ANOVA revealed an effect of group for spike amplitude attenuation (*F*_(2,47)_ = 4.447, *p* = 0.017), and *post hoc* analysis confirmed that spike amplitude attenuation for neurons from HR rats was less than for neurons from naïve rats (*p* = 0.030) and LR rats (*p* = 0.008). Finally, we found that in neurons for all groups, spike amplitude attenuation was correlated with the maximal firing frequency (Pearson’s Correlations: naïve *r* = 0.537, *p* = 0.048; LR *r* = 0.902, *p* < 0.001; HR *r* = 0.692, *p* < 0.001; Figures 2E–G). Thus, the data suggest that the intrinsic excitability of PL-mPFC pyramidal neurons is elevated in HR, but not LR, rats through reduced spike amplitude attenuation during burst firing.

### Norepinephrine Enhances PL-mPFC Neuronal Excitability via cAMP-Dependent sAHP Inhibition

**Figure 3:**
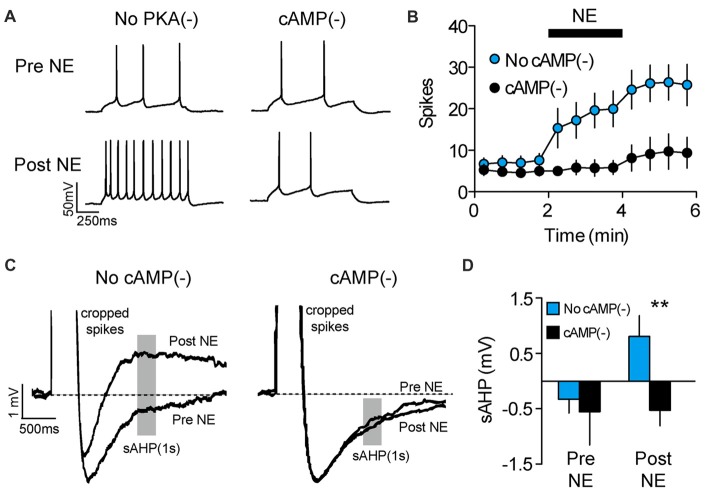
NE enhances PL-mPFC neuronal excitability via cAMP-dependent sAHP inhibition. **(A)** Representative waveforms showing current-evoked action potentials before (top) and after (bottom) application of NE. **(B)** NE increased the number of spikes per sweep, an effect that was blocked by inhibition of cAMP-dependent signaling. **(C)** Representative sAHP waveforms before and after application of NE. **(D)** NE reversed the sAHP into a slow afterdepolarization, an effect that was blocked by inhibition of cAMP-dependent signaling. Line and bar graphs represent the mean ± SEM. NE, norepinephrine; cAMP^(−)^, inhibitor of cAMP-dependent signaling (Rp-2’-O-MB-cAMPs); sAHP, slow afterhyperpolarization; **p < 0.01 vs. control.

In addition to long-term experience-dependent plasticity, intrinsic neuronal excitability could be elevated during memory retrieval through the actions of neuromodulators which act on a shorter timescale. For example, we previously showed that NE rapidly increases the intrinsic excitability of PL-mPFC pyramidal neurons (Otis et al., 2013), although whether NE increases excitability during cue exposure to drive drug-associated memory retrieval is unclear. To address this, we first evaluated the signaling cascade whereby NE adjusts the intrinsic excitability of PL-mPFC pyramidal neurons. Using patch-clamp electrophysiology, we find that bath application of NE rapidly elevates the firing frequency of PL-mPFC pyramidal neurons, an effect that is blocked by Rp-2’-O-MB-cAMPs (Figures 3A,B), the inhibitor of cAMP-dependent processes (specifically PKA and Epac signaling). Two-way ANOVA revealed a time by group interaction (*F*_(11,143)_ = 4.028, *p* < 0.001), and *post hoc* comparisons revealed that NE increased the firing frequency of control neurons (*n* = 8; *p* = 0.003), but not Rp-2’-O-MB-cAMPs-treated neurons (*n* = 7; *p* = 0.331). Next, we evaluated the possible sources for this change in intrinsic excitability. We found that NE reversed the post-burst afterhyperpolarization, revealing a slow afterdepolarizing current (Figures 3C,D) that is consistent with selective inhibition of Ca^2+^-activated K^+^ channels which underlie the sAHP (Wu et al., 2004; Shah et al., 2006). Planned comparisons revealed that NE increased the amplitude of the afterhyperpolarization current in control neurons (*p* = 0.013), but not in Rp-2’-O-MB-cAMPs-treated neurons (*p* = 0.950). In contrast, NE did not significantly influence other canonical mechanisms of intrinsic excitability (Supplementary Table S2). Taken together, NE increases the intrinsic excitability of PL-mPFC pyramidal neurons through cAMP-dependent inhibition of the sAHP. Whether this signaling cascade mediates cocaine-associated memory retrieval, however, is unknown.

### Disruption of cAMP-Dependent sAHP Inhibition in PL-mPFC Persistently Impairs Cocaine Memory Retrieval and Subsequent Reinstatement

**Figure 4:**
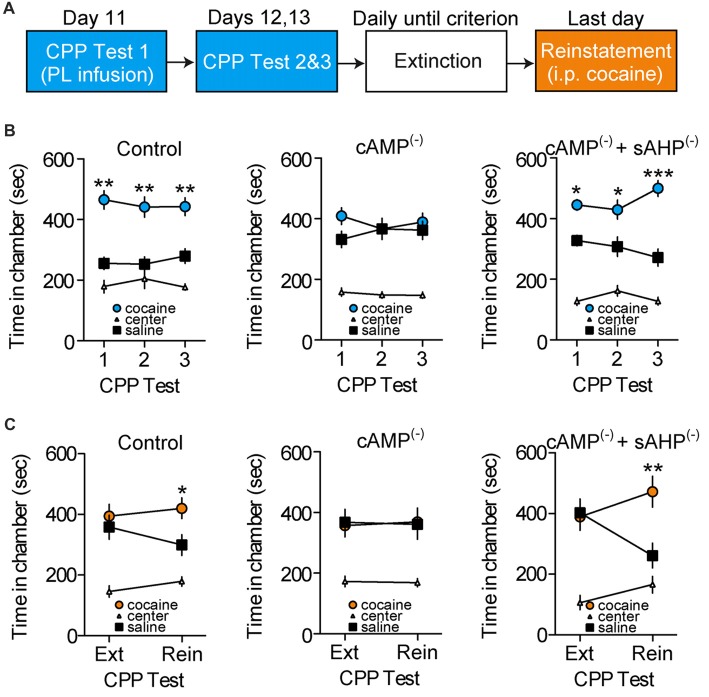
Disruption of cAMP-dependent sAHP inhibition in PL-mPFC persistently impairs cocaine memory retrieval and subsequent reinstatement. **(A)** Schematic showing design of behavioral experiments. Below, the CPP tests are shown in **(B)**, and reinstatement is shown in **(C)**. **(B)** Line graphs revealing that rats given PL-mPFC microinfusions (red arrows) of control (left) or both the cAMP-dependent signaling inhibitor and sAHP inhibitor (right) before the first CPP test only expressed a cocaine CPP across all tests, whereas rats treated with the cAMP inhibitor alone (middle) did not express a cocaine CPP. **(C)** Line graphs showing that rats given PL-mPFC microinfusions of control (left) or the cAMP inhibitor and sAHP inhibitor (right) before the first CPP test only (shown in **B**) expressed cocaine-induced reinstatement following extinction, whereas rats treated with the inhibitor of cAMP-dependent signaling alone (middle) did not express cocaine-induced reinstatement. Line graphs represent the mean ± SEM. cAMP^(−)^, inhibitor of cAMP-dependent signaling (Rp-2’-O-MB-cAMPs); sAHP^(−)^, slow afterhyperpolarization inhibitor (UCL-2077); *p < 0.05, **p < 0.01, and ***p < 0.001 vs. the saline-paired chamber.

We tested whether cAMP-dependent sAHP inhibition is necessary for cocaine-associated memory retrieval. Following conditioning, rats were given repeated CPP retrieval tests (Figure 4A), with PL-mPFC microinfusions of vehicle (*n* = 17), an inhibitor of cAMP-dependent signaling (cAMP^(−)^; *n* = 18 rats), or co-infusions of the cAMP inhibitor and sAHP inhibitor (cAMP^(−)^/sAHP^(−)^; *n* = 11 rats) before the first CPP test only. We found that inhibition of cAMP-dependent signaling blocked CPP memory retrieval during the infusion test, and during subsequent infusion-free tests (Figure 4B). Furthermore, sAHP inhibition reversed this effect (Figure 4B, right panel). Three-way ANOVA revealed a chamber by group interaction (*F*_(4,86)_ = 3.530, *p* = 0.010), and *post hoc* testing for each group revealed that both control and cAMP^(−)^/sAHP^(−)^ rats spent more time in the cocaine- vs. saline-paired chamber during the tests (*p*s < 0.002), whereas cAMP^(−)^ rats did not (*p* > 0.463). Thus, inhibition of cAMP-dependent activity (PKA and Epac signaling) induced a persistent deficit in cocaine-associated memory retrieval, an effect that was reversed by simultaneous sAHP inhibition.

We next examined how inhibition of cAMP-dependent signaling and resulting memory retrieval disruption influences subsequent cocaine-induced reinstatement of the CPP. Following the initial retrieval tests (Figure 4B), rats continued daily CPP tests (also known as extinction tests) until no preference for the cocaine- vs. saline-paired chamber was apparent. Following extinction, rats received a cocaine-induced reinstatement test wherein systemic injections of cocaine were administered before a normal CPP test. We found that control and cAMP^(−)^/sAHP^(−)^ rats showed cocaine-induced reinstatement of the CPP during this test, whereas cAMP^(−)^ rats did not (Figure 4C). ANOVA revealed a chamber by group by test interaction (*F*_(4,86)_ = 2.478, *p* < 0.050). Furthermore, *post hoc* comparisons revealed that no groups expressed a preference for the previously cocaine vs. saline-paired chamber during the last extinction test (*p*s > 0.627). However, while control and cAMP^(−)^/sAHP^(−)^ rats spent significantly more time in the cocaine vs. saline-paired chamber during the reinstatement test (*p*s < 0.019), cAMP^(−)^ rats did not (*p* = 0.916). Taken together, inhibition of cAMP-dependent signaling in PL-mPFC during the first test induced a persistent deficit in cocaine-associated memory retrieval that provided protection against subsequent reinstatement, an effect that was occluded by rescue of sAHP inhibition.

### Disruption of MAPK Signaling in PL-mPFC Has No Effect on Cocaine Memory Retrieval and Subsequent Reinstatement

PL-mPFC cAMP-dependent sAHP inhibition is necessary for maintaining cocaine-associated memory during retrieval, but whether other kinases also maintain memory during retrieval is unclear. Thus, we next assessed the necessity of mitogen-activated kinase (MAPK), a well-known regulator of synaptic plasticity and learning (English and Sweatt, 1997; Di Cristo et al., 2001; Mazzucchelli et al., 2002; Sweatt, 2004; Giovannini, 2006). Following conditioning, rats were given repeated CPP tests, with PL-mPFC microinfusions of vehicle (control; *n* = 11) or a MAPK inhibitor (U0126; *n* = 10) before the first test only. We found that MAPK inhibition had no effect on CPP memory retrieval during the infusion test or during subsequent infusion-free tests (Supplementary Figure S2). ANOVA revealed no chamber by group by test interaction (*F*_(4,16)_ = 0.669, *p* = 0.623) or chamber by group interaction (*F*_(2,18)_ = 1.023, *p* = 0.379), although there was an effect of chamber (*F*_(2, 18)_ = 32.689, *p* < 0.001). Next, *post hoc* comparisons revealed that both control and MAPK^(−)^ rats spent more time in the cocaine vs. saline-paired chambers during the tests (*p*s < 0.005). Thus, PL-mPFC MAPK inhibition did not influence cocaine-associated memory retrieval.

Next, we evaluated how MAPK inhibition during the first CPP test influenced subsequent cocaine-induced reinstatement. Following the initial retrieval tests (Supplementary Figure S2), rats continued daily CPP tests (also known as extinction tests) until no preference for the cocaine vs. saline-paired chamber was apparent. Next, rats were given a cocaine-induced reinstatement test. We found that previous MAPK inhibition had no effect on subsequent cocaine-induced reinstatement of the cocaine CPP. ANOVA revealed no chamber by group by test interaction (*F*_(2,18)_ = 0.253, *p* = 0.779), no chamber by group interaction (*F*_(2, 18)_ = 0.368, *p* = 0.697), although there was an effect of chamber (*F*_(2,18)_ = 43.215, *p* < 0.001). Finally, *post hoc* comparisons revealed that while both groups did not express a preference for the previously cocaine vs. saline-paired chamber during the last extinction test (*p*s > 0.410), both groups expressed a preference during the reinstatement test (*p*s < 0.009). Thus, PL-mPFC MAPK inhibition during the first test had no effect on cocaine-associated memory retrieval or subsequent cocaine-induced reinstatement.

## Discussion

Here, we evaluate how long-term and short-term intrinsic plasticity in PL-mPFC pyramidal neurons may contribute to drug-associated memory retrieval. First, we find that rats exposed to cocaine place conditioning have higher intrinsic excitability in PL-mPFC pyramidal neurons, an effect associated with reduced spike amplitude attenuation during high frequency bursts. Furthermore, we show that both the maximal firing frequency and lack of spike amplitude attenuation positively predict CPP memory retrieval. Next, we find that a PL-mPFC NE-dependent signaling cascade (NE-cAMP-sAHP), which allows short-term enhancement of intrinsic excitability in these neurons, is necessary for CPP memory retrieval. Importantly, inhibition of this cascade causes long-lasting memory impairments which provide protection against subsequent cocaine-induced reinstatement. Taken together, these data reveal that intrinsic plasticity in PL-mPFC pyramidal neurons maintains CPP memory during retrieval.

Our findings reveal that long-term intrinsic plasticity in PL-mPFC pyramidal neurons positively predicts cocaine-associated memory retrieval, although one weakness of our study is that we have not yet defined the underlying channels that contribute to this plasticity. Despite this, previous studies have also shown that cocaine experience results in lasting elevations in the excitability of PL-mPFC pyramidal neurons (Dong et al., 2005; Nasif et al., 2005a,b; Hearing et al., 2013; Sepulveda-Orengo et al., 2018), an effect that is related to elevations in L-type Ca^2+^ channel conductance (Nasif et al., 2005a,b), reductions in whole-cell voltage-gated K^+^ channels (Nasif et al., 2005b) and reduced inward-rectifying K^+^ channels (Dong et al., 2005; Hearing et al., 2013). Voltage-sensitive Ca^2+^ channels shape K^+^-dependent repolarization, afterhyperpolarization and frequency adaptation of action potentials (Meech, 1978; Pineda et al., 1998), and in doing so can prevent spike failure related to spike amplitude attenuation during bursting (Klyachko et al., 2001). Thus, although the specific channel modifications underlying reduced spike amplitude attenuation in PL-mPFC pyramidal neurons from cocaine-treated rats remains unclear, our data support the previously established idea that long-term adaptations in Ca^2+^ and K^+^ channel conductance support high frequency firing in PL-mPFC pyramidal neurons. Furthermore, our data suggest that these particular channel modifications could serve as a mechanism that controls cocaine-associated memory during retrieval.

In addition to long-term intrinsic plasticity, our data suggest that NE-dependent, short-term intrinsic plasticity occurs at the time of cocaine-associated memory retrieval. NE increases the excitability of hippocampal pyramidal neurons (Madison and Nicoll, 1982, 1986; Pedarzani and Storm, 1993) and mPFC pyramidal neurons (Mueller et al., 2008; Otis et al., 2013), specifically through the inhibition of Ca^2+^-activated K^+^ channel conductance which contributes to the sAHP (Madison and Nicoll, 1984; Pedarzani and Storm, 1993). In addition, we previously found that inhibition of PL-mPFC noradrenergic beta-receptors before retrieval disrupts cocaine-associated memories (Otis et al., 2013), identical to what occurs following inhibition of cAMP-dependent signaling (current findings). These data suggest that NE activates beta-receptor dependent cAMP signaling during memory retrieval, leading to elevated intrinsic excitability through inhibition of Ca^2+^-activated K^+^ channel conductance. In further support of this, we now find that rescue of sAHP inhibition, specifically through blockade of Ca^2+^-activated K^+^ channel currents, prevents inhibition of cAMP-dependent signaling from impairing memory retrieval. Collectively, these data reveal that NE-dependent, short-term elevation of PL-mPFC intrinsic neuronal excitability maintains cocaine-associated memories during retrieval.

We find that, at the time of cocaine-associated memory retrieval, PL-mPFC pyramidal neurons have enhanced intrinsic excitability due to both long-term plasticity (reduced spike amplitude attenuation) and short-term plasticity (cAMP signaling). This plasticity is likely to cause adaptations in the activity dynamics of distinct PL-mPFC output neurons, which would lead to circuit-specific changes in downstream neurons. One likely PL-mPFC output circuit is to the NAc core (NAcc), as this pathway is activated by reward-predictive cues, and optogenetic inhibition of this activity reduces conditioned reward-seeking behavior (Otis et al., 2017). In addition, cocaine self-administration leads to an enhancement in PL-mPFC excitatory synaptic drive onto NAcc neurons, and optogenetic inhibition of this plasticity prevents incubation of cocaine seeking (Ma et al., 2014). Additionally, inactivation of PL-mPFC prevents drug-associated cues from driving glutamate release in the NAcc (LaLumiere and Kalivas, 2008), and inhibition of glutamate receptors in NAcc prevents cue-induced reinstatement of drug seeking (Di Ciano and Everitt, 2001; LaLumiere and Kalivas, 2008). Finally, optogenetic inhibition of PL-mPFC cell bodies, NAcc cell bodies, or PL-mPFC terminals in NAcc prevents cue- and cocaine-induced reinstatement of cocaine seeking (Stefanik et al., 2013). Thus, our data showing cocaine-evoked intrinsic plasticity in PL-mPFC pyramidal neurons suggests that PL-mPFC projection neurons, possibly those that innervate the NAcc, are modified following drug experience. However, future studies are needed to identify how drug experience causes intrinsic and synaptic plasticity within precise PFC output circuits to control drug-associated memories.

Our data support a growing body of literature showing that memory can be persistently impaired during retrieval. In rodents, our laboratory has found that one-time systemic, hippocampal, or PL-mPFC injections of noradrenergic beta-receptor antagonists *before* retrieval persistently impairs cocaine CPP memory retrieval and subsequent reinstatement (Otis and Mueller, 2011; Otis et al., 2013, 2014b; Fitzgerald et al., 2016). In contrast, noradrenergic beta-receptor blockade in these regions *after* retrieval do not produce persistent retrieval impairments. Furthermore, optogenetic inhibition of PFC output circuits during retrieval can also persistently impair cue-induced fear, whereas inhibition after retrieval has no effects (Do-Monte et al., 2015). Similarly, in humans, studies reveal that beta-receptor blockade disrupts recall of visual memories and emotional words in normal subjects (Kroes et al., 2010, 2016), and heroin-associated words among human heroin addicts (Zhao et al., 2010). These effects are seemingly long lasting and are irreversible (Kroes et al., 2010, 2016), suggesting a persistent memory retrieval disruption. Furthermore, these studies show that even a single dose of propranolol not only persistently reduces the expression of conditioned fear in humans, but also persistently abolishes fear conditioned responses in the dorsal medial PFC (rodent homolog is PL-mPFC) and hippocampus (Kroes et al., 2016). Thus, work from both human and rodent experiments have striking overlap, supporting the idea that emotional memory expression (i.e., drug and fear memories) can be persistently impaired through inhibition of noradrenergic signaling during retrieval. The data presented here now extend this work and suggest that persistent memory retrieval impairments are related to the inhibition of intrinsic plasticity in PL-mPFC at the time of memory retrieval.

We find that intrinsic plasticity in PL-mPFC controls and maintains cocaine-associated memory retrieval, which could support long-term synaptic plasticity. In support of this, intrinsic excitability allows synaptic signal amplification such that excitatory synaptic events during retrieval will cause elevated spiking in PL-mPFC pyramidal neurons. Conversely, reducing PL-mPFC intrinsic plasticity could block postsynaptic activity during retrieval-related synaptic events, and such asynchrony leads to a form of synaptic depression (spike-timing dependent synaptic depression; Markram et al., 1997; Froemke et al., 2005) that is controlled by noradrenergic signaling (Seol et al., 2007; Huang et al., 2013). Collectively, our data suggest that PL-mPFC intrinsic plasticity allows signal amplification which maintains cocaine-associated synaptic plasticity and memory during retrieval.

## Author Contributions

JO and DM designed experiments, interpreted the data and wrote the manuscript. JO, MF, HY, JB and MD performed the experiments. JO, MF and HY analyzed the data.

## Conflict of Interest Statement

The authors declare that the research was conducted in the absence of any commercial or financial relationships that could be construed as a potential conflict of interest.
