# Supplementary material for: Prefrontal Neuronal Excitability Maintains Cocaine-Associated Memory During Retrieval
Source: Front Behav Neurosci. 2018 Jun 14;12:119. doi: 10.3389/fnbeh.2018.00119 (PMC6010542; doi:10.3389/fnbeh.2018.00119)
Supplement: Supplementary file 1 [file Table_1.DOCX]

**Supplementary Table 1**

| Group | R_N_ (MΩ) | V_Rest_ (mV) | Rheobase (pA) | Threshold (mV) | Amplitude (mV) | Half width (ms) |
| --- | --- | --- | --- | --- | --- | --- |
| Naïve | 194.6 ± 26.9 | -72.1 ± 1.2 | 35.7 ± 6.6 | -52.0 ± 1.7 | 81.2 ± 3.2 | 0.99 ± 0.07 |
| LR | 211.9 ± 19.0 | -71.4 ± 1.5 | 37.9 ± 6.5 | -53.0 ± 0.9 | 85.4 ± 1.9 | 0.97 ± 0.04 |
| HR | 158.5 ± 10.4 | -74.8 ± 3.3 | 36.8 ± 3.3 | -53.2 ± 1.1 | 84.1± 2.3 | 0.99 ± 0.05 |

**Supplementary Table 1 | Basic membrane properties of PL-mPFC pyramidal neurons in naïve, LR and HR rats.** No significant differences were found for any of the above basic membrane properties (for each measurement, one-way ANOVA *p-*values > 0.05). R_N_, input resistance; V_rest_, resting membrane potential. Threshold, amplitude, and half width each refer to action potential properties.
