# Supplementary material for: Prefrontal Neuronal Excitability Maintains Cocaine-Associated Memory During Retrieval
Source: Front Behav Neurosci. 2018 Jun 14;12:119. doi: 10.3389/fnbeh.2018.00119 (PMC6010542; doi:10.3389/fnbeh.2018.00119)
Supplement: Supplementary file 2 [file Table_2.DOCX]

**Supplementary Table 2**

| Group | Time | R_N_ (MΩ) | V_Rest_ (MΩ) | Rheobase (pA) | Threshold (mV) | | Amplitude (mV) | | Half width (ms) |
| --- | --- | --- | --- | --- | --- | --- | --- | --- | --- |
| Control | Baseline | 224.9 ± 34.5 | -71.1 ± 1.5 | 53.8 ± 14.0 | -47.5 ± 1.2 | 75.4 ± 2.8 | | 1.9 ± 0.3 | |
|  | NE | 230.9 ± 29.2 | -65.7 ± 2.9 | 47.5 ± 17.2 | -43.6 ± 3.0 | 72.8 ± 3.2 | | 2.0 ± 0.2 | |
| cAMP^(-)^ | Baseline | 241.9 ± 26.8 | -65.5 ± 1.9 | 47.1 ± 11.9 | -44.7 ± 2.5 | 87.0 ± 2.4 | | 2.8 ± 0.1 | |
|  | NE | 239.9 ± 32.7 | -64.7 ± 1.6 | 45.7 ± 13.6 | -45.4 ± 1.7 | 84.9 ± 3.1 | | 2.9 ± 0.1 | |

**Supplementary Table 2 | Effects of NE on intrinsic excitability of PL-mPFC pyramidal neurons with and without inhibition of cAMP-dependent signaling.** No significant differences were found for any of the above basic membrane properties (for each measurement, effect of time, two-way ANOVA *p-*values > 0.05, time by group interaction, two-way ANOVA *p-*values > 0.05). NE, norepinephrine; R_N_, input resistance; V_rest_, resting membrane potential. Threshold, amplitude, and half width each refer to action potential properties.
