# Supplementary material for: Prefrontal Neuronal Excitability Maintains Cocaine-Associated Memory During Retrieval
Source: Front Behav Neurosci. 2018 Jun 14;12:119. doi: 10.3389/fnbeh.2018.00119 (PMC6010542; doi:10.3389/fnbeh.2018.00119)
Supplement: Supplementary file 3 [file Image_1.PDF]

**Supplementary Figure 1**

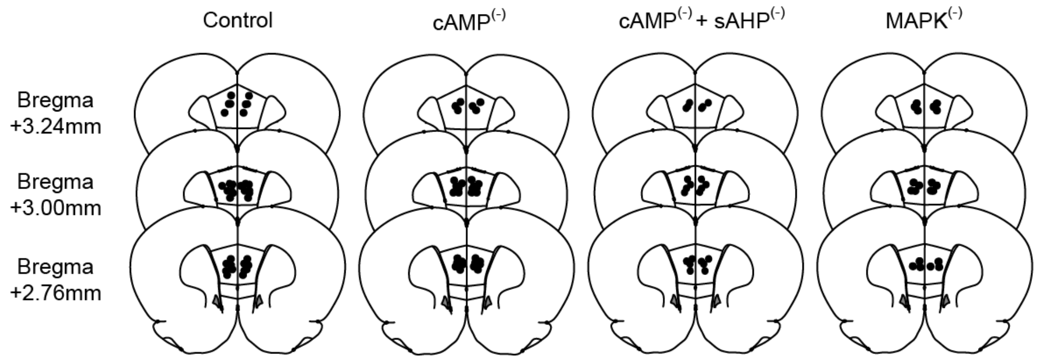


**Supplementary Figure 1 | Histological verification of cannulae placements in PL-mPFC.** Representative drawings revealing microinfusion tip placements (black dots) for all microinjection experiments. MAPK$^{(-)}$, rats treated with MAPK inhibitor (U0126); cAMP$^{(-)}$, rats treated with the inhibitor of cAMP-dependent signaling (Rp-2'-O-MB-cAMPs); sAHP$^{(-)}$, rats treated with slow afterhyperpolarization inhibitor (UCL-2077).
