# Supplementary material for: Prefrontal Neuronal Excitability Maintains Cocaine-Associated Memory During Retrieval
Source: Front Behav Neurosci. 2018 Jun 14;12:119. doi: 10.3389/fnbeh.2018.00119 (PMC6010542; doi:10.3389/fnbeh.2018.00119)
Supplement: Supplementary file 4 [file Image_2.PDF]

Supplementary Figure 2

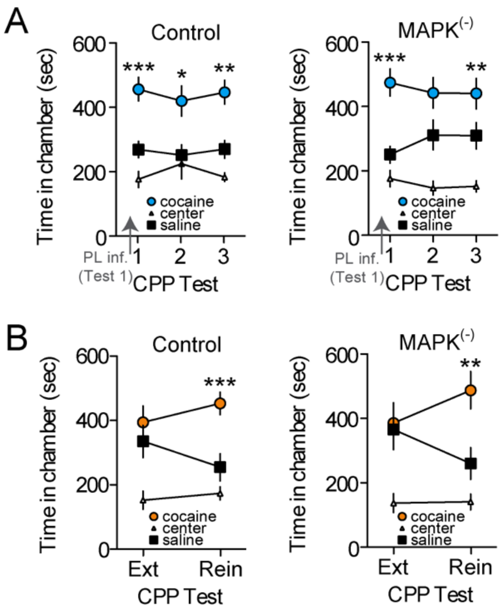

Supplementary Figure 2 | Disruption of MAPK in PL-mPFC has no effect on cocaine memory retrieval or subsequent reinstatement. A) Line graphs revealing that rats given PL-mPFC microinfusions of control (left) or both the MAPK inhibitor (right) before the first CPP test only expressed a cocaine CPP across all tests. **B)** Line graphs showing that rats given PL-mPFC microinfusions of control (left) or the MAPK inhibitor (right) before the first CPP test only (shown in A) expressed cocaine-induced reinstatement following extinction. Line graphs represent the mean ± SEM. MAPK$^{(-)}$, MAPK inhibitor (U0126); *$p < 0.05$, **$p < 0.01$, and ***$p < 0.001$ versus the saline-paired chamber.
